# Experiences and Support Needs of Siblings of Individuals With Prader‐Willi Syndrome‐ Findings From a Two‐Stage Qualitative Study

**DOI:** 10.1111/jar.70122

**Published:** 2025-09-23

**Authors:** Meghana Wadnerkar Kamble, Jen Dawe, Karen Bunning

**Affiliations:** ^1^ School of Health Sciences Faculty of Medicine and Health, University of East Anglia, Norwich Research Park Norwich UK

**Keywords:** experiences, family, parents, Prader‐Willi syndrome (PWS), siblings, support

## Abstract

**Background:**

There is limited evidence exploring sibling's perspective in Prader‐Willi syndrome research.

**Objectives:**

To investigate the experiences and support needs of the siblings of individuals with Prader‐Willi syndrome.

**Methods:**

This two‐stage qualitative study involved siblings aged 11 years onwards (*n* = 11) and parents (*n* = 8). Stage 1 utilised multiple age‐specific focus groups and paired interviews. Data were analysed through integrated discourse and thematic analysis. Stage 2 included consensus meetings utilising the Nominal Group Technique.

**Results:**

Five major themes emerged during Stage 1: family and relationships, food practices, shared understanding, adjustment and advocacy, and support needs. The findings revealed some commonalities in the siblings' discourse, although their experiences varied based on their age. Parents were aware of these differences but had a different focus.

**Conclusions and Implications:**

Sibling's experiences show a developmental trajectory and could explain variable parental perspectives. A lifespan and family‐focused view could help tailor support systems.

**Summary**

- Sibling experiences are dynamic and evolve with age and maturity.
- Considering ‘family’ in a broad sense, including extended family members, might be beneficial in the context of Prader‐Willi syndrome, especially for younger siblings.
- Tailoring advice to the life stages of siblings could be valuable.
- Involving siblings in discussions about the family's needs may positively impact both the individual with Prader‐Willi syndrome and the family system.

## 1 Background

Siblings are frequently involved and even assume caring responsibilities in supporting the family member with a disability (Coyle et al. 2014). Despite their critical role, siblings' perspectives are underrepresented in disability research, which has predominantly relied on parental accounts (Guite et al. 2004) and has seldom adopted a lifespan approach to explore how sibling experiences evolve over time (Hastings 2014). Evidence is equivocal with regards to parents' and siblings' perceptions and experiences of being a caregiver (Guite et al. 2004). This is reported in conditions such as Down syndrome (Cuskelly and Gunn 2003), autism (Rankin et al. 2017), and in rare diseases such as William syndrome (Cebula et al. 2019). Evidence on the impact on siblings is limited in another rare condition, Prader‐Willi syndrome, which is a complex multi‐system neurogenetic condition. Prader‐Willi syndrome is characterised by developmental delays, mild–moderate learning difficulties, life‐long hyperphagia, obsession with food, a multitude of health issues relating to obesity, and accompanying challenging behaviours (Driscoll et al. 2023; Hedgeman et al. 2017). A recent systematic review on experiences of siblings of individuals with Prader‐Willi syndrome reported seven studies (Wadnerkar Kamble et al. 2022; Wadnerkar Kamble accepted). The review found that sibling experiences were influenced by the broader family environment and dynamics more than by the mere presence of a family member with Prader‐Willi syndrome.

When conceptualised through systems theory, a family unit is understood as a social system (Parke et al. 2013) with its own rules and patterns, which can self‐regulate and reorganise, thus taking the emphasis beyond the parent–child dyad, or where parents voice opinions for their children. Family units are interconnected and reciprocal in nature marked by mutual exchange and support (Mauldin and Saxena 2018). According to Family Systems Theory (Bowen 1966), families are made up of codependent subsystems, such as the parent–child and sibling subsystems, and each of these subsystems influences one another. Exploring the sibling experience within the context of family and disability could offer valuable insight into the psychological effects on siblings and the influence of family dynamics, thereby informing effective intervention strategies (Levante et al. 2024; Wadnerkar Kamble accepted). Research has started to consider the role of family systems in intellectual disability (Langley et al. 2021) and in Prader‐Willi syndrome (Mazaheri et al. 2012). However, there is still a tendency to largely focus on parental perceptions to gather the sibling's view (Wadnerkar Kamble accepted).

The act of providing care is generally thought of as unidirectional, i.e., healthy members of the family/unit care for the person with the disability. However, care can be viewed as reciprocal in the context of the family as each member is impacted in some way or the other. Hence, the giving and receiving of care can vacillate from equal to unequal and be rebalanced by the responses of the family members (Dew 2011; Kramer et al. 2013; Meltzer and Kramer 2016). From the sibling's perspective, this could mean the psychological impact of having a family member with a disability, and the extra caring responsibilities or the parentification that can accompany it. From the person with the disability's perspective, this could mean the way their experience of the disability is shaped. From a systemic perspective, it could mean how intellectual and developmental disabilities shape the identities and narrative in a family unit, and the way developmental changes can impact the experiences of giving and receiving care over time. For example, having an older sibling with Prader‐Willi syndrome and the presence of behavioural difficulties is known to cause more stress for the younger sibling (O’Neill and Murray 2016).

There is limited evidence of sibling perspectives in Prader‐Willi syndrome research, which contrasts with the advice provided by voluntary sector organisations to support siblings (PWSA UK 2022; IPWSO 2023). In the context of rare conditions such as Prader‐Willi syndrome, the experiences of siblings are often overlooked—partly due to limited public awareness and a lack of available support. Gaining a deeper understanding of siblings' perspectives in these specific syndromes is essential for meeting family support needs and addressing the marginalisation these families may face (Marquis et al. 2023). Taking the sibling‐centric view and considering the whole family approach is essential for designing appropriate interventions to address the family's support needs and to appropriately support the person with Prader‐Willi syndrome. There is a case for taking a lifespan perspective on the changing dynamics of care in Prader‐Willi syndrome. It is also important to consider how siblings' support needs evolve over time. For instance, younger siblings may benefit from clear information about their brother or sister's disability, emotional support, and help managing difficult feelings. In contrast, adult siblings might require access to peer support networks and practical guidance for caregiving and planning (Halm and Arnold 2017).

This study used the family systems theory (Bowen 1966) as a framework to guide the research enquiry. This study addressed the evidence gap by taking an in‐depth understanding of sibling experiences from a lifespan perspective by interviewing siblings across the age groups and understanding the parental perspective in relation to the siblings.

## 2 Aims and Research Questions

This study aimed to investigate the discourses and personal narratives around the lived experience of having a sibling with Prader‐Willi syndrome and to understand the support needs of the siblings. This study also sought the parental perspective and support needs in relation to the siblings.

The research questions (RQs) guiding this investigation were:

What are the experiences of growing up and family life of siblings who have a brother or sister with Prader‐Willi syndrome?

What are parents' perceptions of their child's experience of growing up with a sibling who has Prader‐Willi syndrome?

What are the support needs of the siblings in terms of priority and course of action?

What are the support needs from the parent's point of view?

## 3 Methods

### 3.1 Study Design

This study was designed as a two‐stage, exploratory qualitative study, where Stage 1 findings informed Stage 2. The exploratory design was used to seek a deeper understanding of the sibling experience to be better able to position the findings to address the research gap. Stage 1 utilised virtual focus groups or paired interviews to get an in‐depth account of the participants' realities (Miller and Glassner 2016) pertaining to RQs 1 and 2. Stage 2 utilised the Nominal Group Technique in a virtual form (Delbecq et al. 1975; McMillan et al. 2016) to gather consensus on the support needs pertaining to RQs 3 and 4.

### 3.2 Participants

Siblings aged 11 years and above, either living at home with their brother/sister with Prader‐Willi syndrome or having left home, were included. From the age of 11 years, most children can engage with semi‐structured interviews owing to the cognitive developmental stage (Christensen and James 2008). Parents/primary carers with day‐to‐day caring responsibility for the child with Prader‐Willi syndrome and who had at least one child without Prader‐Willi syndrome were included. Participants were excluded if they self‐identified as having health and/or psychological needs that, in their view, could impact on research participation, and/or lacking the mental capacity for informed consent. Siblings were grouped based on age into three groups: younger siblings: 11–13 years (group one) and 14–17 years (group two), and older siblings: 17 years+ (group three) (siblings *n* = 11). Parents made the fourth group (*n* = 8) making a combined sample size of 19 participants spread across 3 focus groups and 4 paired interviews, i.e., seven units of analysis (Table 1). Participation in the study did not require siblings and parents to be from the same family unit. However, one parent–child pair—a mother and her daughter (aged 11–13 years) was included. Although recruitment was open to participants beyond the UK, all individuals who took part were UK‐based. The sample lacked ethnic diversity, with only one sibling of British Asian origin, despite receiving expressions of interest from a demographically diverse pool of potential participants. Stage 2 included six participants from Stage 1: four older siblings and two parents. However, only one parent was able to stay for the entire session.

**TABLE 1:** Participant demographics.

Group	*N* for Stage1	Range and (Average age)	Gender	Family member with Prader‐Willi Syndrome	Any other siblings/children	Number of focus groups/paired interviews Stage 1	*N* for Stage 2
Group 1: Siblings 11–13 years	2	11–12 years.; (11.05 years)	Female	2: 1 sister, 1 brother	None	1 paired interview	n/a
Group 2: Siblings 14–17 years	2	14–15 years.; (14.05 years)	1 gender fluid, 1 female	2: both sisters	None	1 paired interview	n/a
Group 3: Siblings 17+ years	7	27–60 years.; (43.05 years)	2 males, 5 females	7: 6 sisters, 1 brother	1 brother	1 focus group and 1 paired interview	4
Group 4: Parents	8	40–57 years.; (50 years)	Mother	8: One child each with Prader‐Willi syndrome	1–2 unaffected children per parent. Total 5 daughters, 6 sons	2 focus groups and 1 paired interview	2

### 3.3 Ethical Considerations

Ethics approval was received from the Faculty of Medicine and Health, University of Eat Anglia, UK, November 2022 (ETH2122‐1929). Participants had the right to withdraw from the study at any stage without giving reasons. There were no withdrawals in the study or any adverse events. Pseudoanonymisation was used where each participant was assigned an alpha‐numeric participant ID, e.g., FG1P1, to indicate their focus group number. This helped during the data analysis to see any commonalities/differences across the groups. Participants' personal information was separated out and securely stored in a bespoke Excel spreadsheet on the first author's (MWK) secure University device. Participants were instructed to join the virtual platform using the preassigned pseudonym, reminded to keep their camera off and not to disclose any personally identifiable information during the online interviews. To uphold ethical standards and promote participant comfort and engagement, particularly given the sensitive nature of the research topic, participants were invited to join the session with their cameras turned off. This approach aimed to enhance anonymity and reduce potential social desirability bias (Lewis and Muzzy 2020; Brown 2022). In contrast, the facilitator(s) kept their cameras on to maintain a sense of presence and support during the session. Any identifiable information was removed or anonymised from the transcripts. The first two authors (MWK and JD) had access to the data.

### 3.4 Recruitment and Sampling

Age‐appropriate promotional flyers and study information packs were created for each group. The study was advertised across several regional and national charitable organisations, such as Prader‐Willi Syndrome Association UK and Sibs. Additionally, the flyers were disseminated via the University's social media pages. Convenience snowball sampling, as a non‐probability sampling method suitable for studies of persons with rare traits, was used (Galloway 2005). Participants were requested to share the flyers with their own family members and relevant contacts.

### 3.5 Procedure for Recruitment

On their request, participants were emailed a study information sheet and consent form, which covered both stages of the study. Participants were contacted 48 h later to answer any queries and to check the inclusion criteria. Following this, eligible participants, or their parents (when under 16) were sent the consent form. Younger siblings under 16 years of age gave assent, and their parents gave consent.

### 3.6 Data Collection

Following the consent, participants were contacted to arrange the focus groups/paired interviews. Participants joined the specific online focus groups on Microsoft Teams. The interviews were recorded and auto transcribed using the in‐built functionality of Teams. Data were collected over December 2022 to March 2023.

Stage 1 used an interview guide (Appendix A.1) to inform the semi‐structured questions, which were developed through a familial lens (Rosenblatt and Fischer 1993). The interview questions were developed to explore the dynamics within the family system, focusing on three key sub‐systems: the sibling with Prader‐Willi syndrome, the siblings participating in the interviews, and the broader context of family life, including the home environment and parental roles. The aim was to understand the siblings' perspectives on their family experiences and their views of their brother or sister with Prader‐Willi syndrome, as well as to identify the types of support they consider important. Additionally, parents were invited to share their perceptions of how their child without Prader‐Willi syndrome experiences growing up alongside a sibling who has the condition. Six focus groups/paired interviews were facilitated by the second author (JD) and one paired interview was facilitated by the first author (MWK).

Stage 2 used the Nominal Group Technique (Delbecq et al. 1975; McMillan et al. 2016) to prioritise the support needs from those shared during Stage 1, and to find ideal ways to achieve these. The first author (MWK) has prior training and experience in using the Nominal Group Technique (NGT) from previous research projects. As part of the preparatory phase for this study, the first author conducted three one‐hour training sessions with the second author (JD). These sessions covered an overview of the NGT process, facilitation strategies, and the technical aspects of the digital setup. Both authors collaboratively tested the digital platform to ensure smooth implementation. JD also engaged in wide reading on NGT to deepen their understanding of the method. JD, who has strong skills in facilitating group discussions, effectively applied these abilities during the NGT sessions.

The University's online platform ‘Blackboard Collaborate’ was used for its functionality to run polls and invite audience participation whilst preserving their anonymity. The meeting started with an example slide to illustrate the voting process and to explain the digital tools used for this purpose, which the participants could practice (Appendix A.2). Participants were then presented with paraphrased statements based on the thematic analysis of the transcripts produced during Stage 1. Participants were asked to review the statements and vote on their top five choices (Appendix A.3).

### 3.7 Data Analysis

**FIGURE 1:**
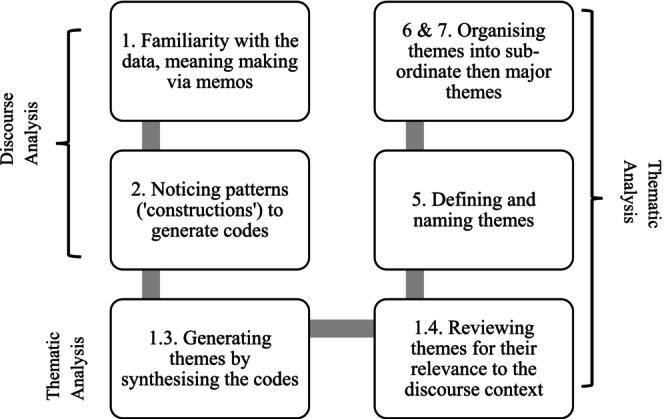
Illustration of the analytic process.

Stage 1 had seven units of analysis. Transcripts were checked for accuracy with audio recordings. Data were analysed by the second author (JD) using NVivo (version 14) and cross‐checked by the first author (MWK). Stage 1 used an integrated approach of discourse (Potter and Wetherell 1987) and interpretive thematic analysis (Braun and Clarke 2006) to gain a deeper understanding of how the participants perceive their reality. The discourse analysis was guided by a social constructionist framework (Potter and Wetherell 1987), enabling a meso‐ and micro‐level examination of how participants constructed their understandings of family and social life through language. This approach focused on the meanings embedded in their word choices and expressions. This was followed by thematic analysis (Braun and Clarke 2006), which was employed to identify recurring themes within the data, capturing the underlying meanings and assumptions that shaped participants' experiences and perspectives. Thus, the discourse was used to shape and interpret the themes (Figure 1). Transcripts were reviewed at every stage and relevance to the research question confirmed. Unit‐level analyses allowed presenting a comparative picture and to notice the dominant discourse in each group. There were several commonalities across the individual groups. Hence, it was decided to present these together as an additional overall thematic narration informed by the discourse.

In Stage 2, all votes were totalled and a list comprising the top five most highly voted support needs was compiled.

### 3.8 Rigour and Positionality of the Researchers

The first two authors (MWK and JD) considered their positionality as researchers (Ryen 2016) by using reflexive practice, records, and an audit trail. This included the researchers' purposefully positioning the participants as holding expertise during the interviews and by introducing themselves as facilitator(s). Stage 2 involved the participants providing member checks about the findings through discussion and voting during the consensus meeting, further assuring the credibility of the study findings. Triangulation was used in all stages of analyses, ensuring the credibility, confirmability, dependability, and transferability of the findings. This paper includes participant quotes and a description of findings to further ‘evidence’ quality (Tracy 2010).

Additional reflections were undertaken regarding the authors' positionality, particularly in relation to their connections with Prader‐Willi syndrome. The first (MWK) and the senior authors (KB) have worked clinically with individuals with Prader‐Willi syndrome. In addition, KB has specific experience of providing regular respite care for an adult with Prader‐Willi syndrome. Authors come from a diverse background and bring personal experience as caregivers in complex care contexts. These backgrounds informed the research focus of the first author and contributed to shaping the study's direction. Collectively, the authors bring a balance of objectivity and empathy essential for conducting research involving complex and rare conditions such as Prader‐Willi syndrome.

## 4 Findings

### 4.1 Stage 1 Findings: Overall Thematic Illustration

**FIGURE 2:**
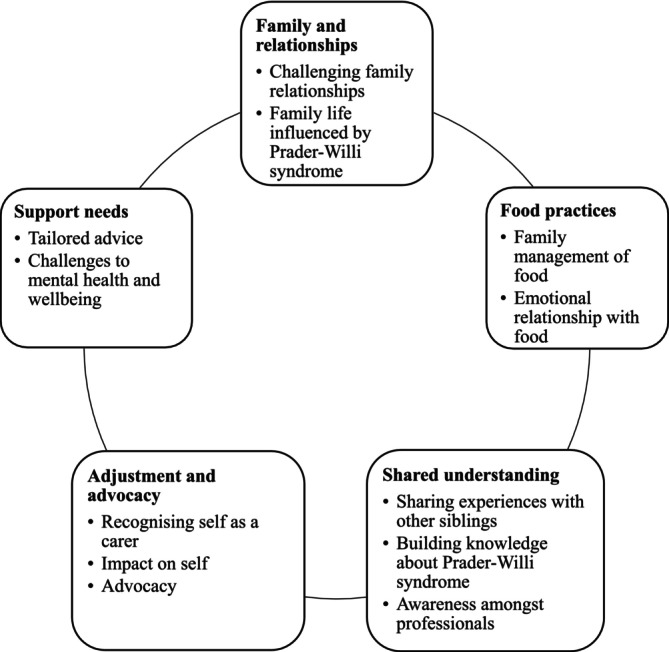
Thematic representation.

Five major themes emerged at Stage 1 (Figure 2), i.e., Family and relationships, Food practices, Shared understanding, Adjustment and advocacy, and Support needs. Each major theme consisted of two to three subordinate themes.

#### 4.1.1 Major Theme 1 Family and Relationships

This theme contained discourse on family relations and life, which also made the subordinate themes.

##### 4.1.1.1 Challenging Family Relationships

A prominent discourse across all the age groups was the lack of equitable treatment by parents. Parents showed awareness of this, and said it was difficult to manage as they had to parent in different ways. Older siblings reminisced about gradual deterioration in the relationship with their brother/sister with Prader‐Willi syndrome and the breaking of their parents' marriage due to their fathers not being able to cope with the child with Prader‐Willi syndrome. Relationships with the extended family, such as with grandparents, were viewed as having a positive ‘buffering’ impact by the younger two groups but viewed as tricky by older siblings and parents due to conflict of practices around food and family routines.

Yeah, because we can do the same thing and they'll Always get into less trouble [for doing that]. Actually we can get into trouble for punishing her too hard because she is disabled or whatever. (Younger sibling group one, 11‐13 years, FG6P1)

##### 4.1.1.2 Family Life Influenced by Prader‐Willi Syndrome

Most siblings across the age groups spoke about coping strategies, such as avoidance, distraction, and parental mediation to cope with behavioural difficulties and to navigate family life around their brother/sister with Prader‐Willi syndrome. Parents, especially of older children, were concerned that their child would not want a family of their own. This was supported in the narrative of some older siblings. Those older siblings who did go on to start their own family worried about the genetic risk and the relationship of their spouse and children with the sibling with Prader‐Willi syndrome.

Uh, and if my sister wasn't already at home when I got home 'd have a chat about my day very, very quickly because as soon as my sister was there, it was It was very difficult to have a conversation At all. (Older sibling group three, 17+ years, FG5P2)

#### 4.1.2 Major Theme 2 Food Practices

This theme was based on the discourse around food and its impact on the sibling's life. Two subordinate themes made up this major theme as seen from across the age groups, i.e., family management of food and relationship with food.

##### 4.1.2.1 Family Management of Food

This theme covers how issues around food/eating were managed, owing to the challenges posed by the compulsive food‐related actions of their brother or sister with Prader‐Willi syndrome. Younger siblings from group one spoke about food in ‘matter of fact’ terms but shared instances of mealtimes being tricky. Older siblings reflected on limited access to kitchen/freezer whilst growing up.

And I remember the tantrums and the stubbornness and the food stealing and the locked doors and the freezer. Even when she stole the frozen baguette and ate the whole thing, and we had to start locking the freezer. (Older sibling group three, 17+ years, FG3P1)

##### 4.1.2.2 Emotional Relationship With Food

Younger siblings from group two equated food to emotions, mostly citing their feelings of 'guilt' around eating food that their brother/sister with Prader‐Willi syndrome could not have. Older siblings spoke about sporadic eating habits, not understanding they could eat food when they wanted it once they moved out of their family home, stating they were very familiar with feeling hungry and seeking psychotherapeutic counselling to help them understand how they deal with food.

But uh, I'm very used to being hungry. If I'm hungry, I won't say anything. If I'm hungry, I won't also do anything about it. Not in like….a self harming sort of measure, but it's just something I got used to do. You know if you're hungry, don't say anything … (Older sibling group three, 17+ years, FG5P2)

#### 4.1.3 Major Theme 3 Shared Understanding

This major theme was based on sharing own experiences and spreading awareness about Prader‐Willi syndrome. Three subordinate themes, i.e., sharing experiences, building knowledge, and awareness amongst professionals made this theme.

##### 4.1.3.1 Sharing Experiences With Other Siblings

The siblings and parents spoke about how glad they were to speak with others in a similar position and to share experiences. For the older siblings, this study was the first chance they had to speak to others in a similar position. Siblings acknowledged that sharing experiences should happen naturally in Prader‐Willi syndrome related events and other generic events not focused on Prader‐Willi syndrome. This notion was shared by the parents.

Yeah, it's. Yes, this this so different. So it's good to actually – oh I don't feel so alone. (Older sibling group three, 17+ years, FG5P3)

##### 4.1.3.2 Building Knowledge About Prader‐Willi Syndrome

Almost all siblings spoke about self‐directed or guided efforts to educate themselves about Prader‐Willi syndrome. In some instances, this helped them understand why their sibling with Prader‐Willi syndrome behaved the way they did.

I remember like maybe a year ago or some months ago, my sister had a really, really big tantrum like probably the biggest one and something that my dad did is he showed me this, this document, I think that was given to him by the hospital or by some doctor who just who was, um the document talked about how to take care of them, but also handle them while they're having tantrums out or just with day‐to‐day life with food and things like that. And seeing that was incredibly helpful. (Younger sibling group two, 14–17 years, FG1P2)

##### 4.1.3.3 Awareness Amongst Professionals

This theme reflected the frustration regarding the limited or lack of awareness in medical professionals, service providers, and educators about Prader‐Willi syndrome. For example, not understanding the condition well enough, not considering the feelings and experiences of the person with Prader‐Willi syndrome, and the carer role of siblings or parents.

….And if you can't even turn to GPs and health workers, my sister at the moment is having terrible trouble with her teeth….it's made us realize ….that the profession itself is just not clear on, you know, on needs and understanding…. (Older sibling group three, 17+ years, FG3P1)

#### 4.1.4 Major Theme 4 Adjustment and Advocacy

This theme was based on the practical and psychological accommodations and adjustments made by the participants. This major theme was made of three subordinate themes, i.e., recognising self as a carer, impact on self and advocacy.

##### 4.1.4.1 Recognising Self as a Carer

This theme comprised of participants' conceptualisation as informal carers. The older siblings reflected on taking over caring responsibilities from their parents, inadequate resources to prepare them for both the emotional and practical (e.g., financial and legal) aspects of being a primary carer and preparing the brother/sister with Prader‐Willi syndrome for this eventuality. Interestingly, younger siblings in group two, i.e., 14–17 years, defined themselves as being carers, whilst siblings in group one, i.e., 11–13 years, did not. However, these siblings described undertaking activities that could be understood as caring, such as issuing injections to their sibling or speaking with the medical practitioners. Parents frequently shared concern about their children becoming primary carers when older.

Yeah, I'd say I'm a carer for her and I do a lot of jobs for her on a day‐to‐day basis. (Younger sibling group two, 14–17 years, *FG1P3)*

##### 4.1.4.2 Impact on Self

This theme comprised how having a sibling/child with Prader‐Willi syndrome shaped the persona of the participants. Younger siblings from group two and older siblings reflected that the focus on the brother/sister with Prader‐Willi syndrome made it difficult for them to understand their place within the family. The siblings also described feeling 'second place.' The parents shared awareness and concern about this.

in the house she does get a lot more attention because of her needs and if she wants something my parents have to like, go to her first so she doesn't like have a tantrum or like get cross and do something bad. (Younger sibling group two, 14–17 years, FG7P3)

Siblings reflected that they have many friends who had some form of atypicality. Parental discourse supports this reflection. Younger siblings from group two and some older siblings described considering or having opted for a caring profession, such as in healthcare and counsellors. The parents also described changing own careers, e.g., to work with children with additional needs.

##### 4.1.4.3 Advocacy

This theme was based on the instances of families making a case for the rights of the sibling/child with Prader‐Willi syndrome. Several of the siblings, across all sibling groups but mainly groups one and three, described being an advocate for their brother/sister with Prader‐Willi syndrome. This included speaking at conferences for families of people with Prader‐Willi syndrome and at their schools, telling their friends about Prader‐Willi syndrome as a condition. The youngest siblings from group one regarded being active advocates as a usual family responsibility. The older siblings advocated in terms of practical, financial, and legal concerns.

PWS is one that a lot of people should know about because it's a really big one………. I think it be a benefit to know….what happens if someone has Prader Willi syndrome and ….how other people like relatives go through it. (Younger sibling group one, 11–13 years, FG6P2)

#### 4.1.5 Major Theme 5 Support Needs

This major theme was based on the importance of appropriate and individualised advice and mental health needs. Two subordinate themes, i.e., tailored advice and mental health and well‐being, made this theme.

##### 4.1.5.1 Tailored Advice

There was a widely shared view that guidance should be tailored to the unique needs of each family and sibling. Many younger siblings had interacted with peers in similar situations, while others found support from family and friends to be particularly helpful. Preferences for meeting formats varied by age, with older siblings favouring in‐person interactions.

go to your parent or carer to see if they will help you or give you advice and they will like obviously help you out anyway… (Younger sibling group two, 14–17 years, FG1P3)

##### 4.1.5.2 Challenges to Mental Health and Well‐Being

This theme contained the positive and negative emotional impact, guilt, and the complexity of the emotional experience. All groups described their daily life as having extreme emotions depending on the person with Prader‐Willi syndrome and their behaviour. However, all siblings shared a positive affinity for their brother/sister with Prader‐Willi syndrome and broader family life. Siblings sought help for their mental health and wellbeing via schools, parents, psychotherapy, and counselling. The parents shared concerns for their child's mental health and wellbeing, but mainly due to food management and secretive communicating practices.

I've got all of these, like flashbulb memories of these really nice things that we were doing [followed by] this flashbulb of the bad thing that happened. I was just in this constant fear of what's gonna happen. When is there gonna be an explosion and that still gets me now. I'm just always thinking, well, what she's gonna do, what's gonna happen. You're always on edge. (Older sibling group three, 17+ years, FG5P2)

### 4.2 Comparing and Contrasting the Group Specific Findings

#### 4.2.1 Group 1: Younger Siblings Aged 11–13 Years

Siblings actively described behaviours that could be considered as advocacy and spoke passionately about the rights of people with Prader‐Willi syndrome. Siblings shared negative aspects of having a brother/sister with Prader‐Willi syndrome but were equally open to sharing their family life with friends, recognised the parental efforts, had met other families caring for someone with Prader‐Willi syndrome but did not willingly mention food or share any challenges in relation to eating. Their favourite way to meet people was via fun events such as outdoor activities and parties. This contrasted with the older siblings who kept their friends ‘separate’ from the family home, ‘compartmentalising’ different relationships in their lives, and shared an emotional relationship with food.

#### 4.2.2 Group 2: Younger Siblings Aged 14–17 Years

The notion of siblings taking ‘second place’ to their brother/sister with Prader‐Willi syndrome was prominent in this group. This group reflected on the difficulties faced by their parents and were sympathetic to the challenges that their brother/sister with Prader‐Willi syndrome faced. Unlike group one, this group described their brother/sister with Prader‐Willi syndrome in purely positive terms. Like group three, these siblings spoke about developmental maturity in their relationship with their sibling with Prader‐Willi syndrome and adjusting to the situation as a coping strategy to deal with challenging behaviour. These siblings also described themselves as being ‘carers’.

#### 4.2.3 Group 3: Older Siblings Aged 17 Years+

This group spoke about the importance of sharing experiences with others in similar situations. They reflected on the limited knowledge of Prader‐Willi syndrome in the general population, which made it harder for them to talk about their own experiences. Unlike group one, this group spoke at length about their relationship to food as not being normal. This group described a gradual deterioration in their relationship with their brother/sister with Prader‐Willi syndrome. Some of the siblings also shared about acting as mediators between their parents and their sibling with Prader‐Willi syndrome, and about the deterioration in their parents' relationship.

#### 4.2.4 Group 4: Parents and Carers

The discourse by parents mirrored some of the aspects shared by the siblings but had a different focus. Much of the discussion by parents involved concerns about the effect of food management on their child who does not have Prader‐Willi syndrome, especially if that child was a female. The parents also shared concerns about their other children in terms of their social lives and them becoming the primary carer. Like older siblings, the parents also described feeling isolated. The parents expressed guilt and sadness about asking their child(ren) to adapt their behaviour to appease their brother/sister with Prader‐Willi syndrome. The parents described their family lives as containing extremes of emotions, which they felt may be different to how other families experienced their daily lives.

### 4.3 Summary From Stage 1

Findings highlight the specific ways that the siblings' experience family life with a brother/sister with Prader‐Willi syndrome. It is important to emphasise here the interconnectedness of the themes, i.e., all groups reported similar findings, including awareness of challenges faced by other members of the family. Interestingly, findings reveal a potential developmental trajectory where the youngest group (group one) is invested in advocacy, which shifts into adjustments in the adolescent years (group two) and moves into sharing of experiences in later years (group three).

### 4.4 Stage 2 Findings

The top five ranked support needs from the older sibling's consensus group were centred around having access to information and support networks. Parents shared these needs but also expressed a need for support with managing parenting practices (Table 2). In the absence of consensus data from the younger groups, these can not be interpreted for the younger siblings group. However, the similarities in the themes across the groups are indicative that at least some of these priorities could be applicable for the younger groups.

**TABLE 2:** Top five support needs.

#	Siblings	Parents
1	More information should be provided for adult siblings about health concerns experienced by their own adult sibling who has Prader‐Willi syndrome.	More childcare and/or respite for parents and carers of younger children.
2	Health, education, and other professionals, should have more training and knowledge about Prader‐Willi syndrome, including how it is distinct to other neurogenetic conditions.	Counsellors should be trained in Prader‐Willi syndrome and family life.
3	There should be (more) opportunities to meet other siblings anonymously (e.g., online).	Need for advice about parenting siblings in different ways.
4	Research about Prader‐Willi syndrome should be better communicated to siblings‐both in terms of how it is written and where it can be found.	More information for siblings of all ages about having a brother or sister who has Prader‐Willi syndrome.
5	Siblings of all ages should be offered (free or affordable) counselling with therapists who have specialist knowledge of Prader‐Willi syndrome and its impact on the siblings.	More opportunities for parents and carers of children with Prader‐Willi syndrome to meet, share experiences and support.

## 5 Discussion

### 5.1 Experiences of Siblings

The impact of having a sibling with Prader‐Willi syndrome changed with age and maturity, such that the relationship with their brother/sister with Prader‐Willi syndrome got better or worse depending on the age of the sibling and the person with Prader‐Willi syndrome. While siblings were engaged in informal caregiving from a young age, the conscious recognition and identification of themselves as carers appeared to emerge progressively from adolescence onward. Some older siblings appeared to channel their experiences into career choices related to caregiving, while younger siblings often extended their caring roles into their peer relationships. This was in addition to siblings accessing professional interventions for themselves, such as psychotherapy and counselling. This could be because the existing interventions do not fulfil the need for tailored and individualised support (Wolff et al. 2023), especially in the case of Prader‐Willi syndrome. Experiences of the impact of food management practices, and a need to connect and expand on support networks showed a developmental trend. This may be indicative of changing priorities and needs as the sibling matures. Sibling relationships are lifelong. Therefore, a lifespan perspective on sibling research is needed to understand sibling experiences over time through childhood and into adulthood (Hastings 2014), which this study aimed to do. A shift in focus across the age groups from advocacy and adjustment in the younger groups to a need for connections in later years points towards the developmental and dynamic impact of having a brother/sister with Prader‐Willi syndrome.

Whilst siblings reflected on the lack of equitable treatment by their parents, they also learnt from their family environments. Siblings developed behaviours and characteristics, such as putting themselves in the background to respond to the environment and reduce parental burden. This could also be evident of the sibling subsystem and the family unit self‐regulating and reorganising itself. Family units which adapt and evolve in response to stressors are better able to cope with demanding circumstances (Blacher and Baker 2007). Siblings' response is dynamic and shaped by the environment within and external to the family. For example, the role of the extended family members was viewed differently by the younger and the older groups as more or less helpful. This narrative also partly came across as shaped by the parents. This could be evident of the interconnectedness and impact of the parent–child and sibling subsystems on each other (Zemp et al. 2021). Reflections from the older siblings about their early experiences shaping their own family life as a grown‐up point to long lasting impact (Kirk and Pryjmachuk 2024) and concur with the intergenerational transmission of attachment and relational patterns (van IJzendoorn and Bakermans‐Kranenburg 2019).

### 5.2 Parent's Perception

The parents showed an awareness of the sibling experiences, but their focus was different. At times there was a differential focus in the case of mental health, whereby the parents' shared worries about 'deception' about food, whereas the siblings had a focus on complex emotions. The parents were more concerned about the impact of food management and social isolation on the sibling. Whereas the siblings were more focused on the emotional impact of food management practices. This discrepancy aligns with previous findings highlighting differences in sibling and parent reports in the context of chronic illness and disability (Guite et al. 2004). It is particularly pertinent in the case of Prader‐Willi syndrome, where hyperphagia and the associated food management routines often dominate family life and structured care (Currie et al. 2024a). Such routines may inadvertently obscure the distinct impact these caregiving practices have on siblings. Parental attempts at parenting differently were viewed as non‐optimal by the siblings and could be adding to the complex emotions experienced by the siblings. For example, parental behaviours of secretive communicating practices when the child with Prader‐Willi syndrome was present (Kowal et al. 2022) were deemed to be shaping the guilt experienced by the siblings. This also exemplifies the far‐reaching impact of Prader‐Willi syndrome on family life, highlighting the delicate balancing act parents must perform daily to maintain equitable parenting across their children (Currie et al. 2024b). Overall, parents showed an awareness and concern about the potential future role of their child as a carer, but their priorities were coloured by their life circumstances and not always aligned with those of the siblings (Mazaheri et al. 2012; Dew 2011). This mimics the context of children's mental health where parents had a high threshold for perceiving mental health needs in their children (McGinnis et al. 2022).

### 5.3 Support Needs of the Siblings

Family was seen as a support base. Having at least one person in the immediate or extended family as an ally was important. Indeed, in the case of having a family member with a disability, having individuals to share the caregiving or emotional burden acts as a protective factor (Rakap and Vural‐Batik 2024). For the older siblings, it was a long journey to accommodate and adjust to being a sibling of someone with Prader‐Willi syndrome, with professional help coming much later. This could reflect the differences in the knowledge, attitude, and availability of appropriate professional support services, such as counselling, in the present and past. A need is evident for individualised advice tailored to the life course of the siblings. This was mirrored in Stage 2, whereby practical advice was needed on the health of the brother/sister with Prader‐Willi syndrome, advice for professionals, shared knowledge and experiences, mental health, and wellbeing for siblings. The practical nature of these support needs is not surprising, considering the consensus meetings had the older siblings who shared a solution‐focused view. However, interconnectedness between the categories indicates that effectively addressing one priority could have a positive impact on other aspects.

### 5.4 Parents View of Support Needs

Like parent's perceptions, parents view of support needs was informed by practical concerns. Parents also shared a need for support with parenting practices. This could be because parents could be parenting in different ways, adopting variable parenting practices to cater to the needs of the disabled and non‐disabled child, which could be vastly different to a family where there is no disability. Parenting a child with a disability is known to contribute to parenting stress but there is limited evidence in relation to any variability in parenting practices in families caring for a child with a disability and a typically developing child (Raya et al. 2013).

### 5.5 Reciprocity, Care, and Agency

Historically, the narrative around care and disability has portrayed the person with the disability as at the receiving end of care and lacking in agency (Morris 1997). However, the interconnected and reciprocal nature of the family systems could mean that each member of the family, including the person with Prader‐Willi syndrome, allows an opportunity for the system to evolve and adapt (Dew 2011; Kramer et al. 2013; Meltzer and Kramer 2016). This was evident in the data whereby the older siblings shared positive aspects of their brother/sister with Prader‐Willi syndrome and reflected on the impact that had on their own life.

#### 5.5.1 Limitations of the Study

Online interviews proved challenging with the youngest of siblings who needed more probe questions. The camera off mode meant that the facilitators had no access to any visual cues from the participants. This was mitigated by briefing the participants on using the ‘raise hand’ function of the virtual platform. However, the analytic methods used enabled consideration of meaning beyond the ‘surface level’ by combining the interviews context and the external context, i.e., the family unit. Most of the participants were female, which is consistent with a trend seen in other studies on Prader‐Willi syndrome and parents/siblings. Although the sample size was limited, the study offers a rich and detailed account supported by a rigorous methodological approach. Stage 2 findings need to be interpreted with caution in terms of younger siblings.

## 6 Conclusions

This qualitative study provides an integrated view of the siblings and parent's perspective and illustrates the developmental trajectory of the siblings' evolving understanding. Findings explain the nuances behind equivocal results from siblings in terms of the negative and positive factors and the differences between the parental and sibling perspectives. Having the lifespan and family‐focused view is essential to tailor support systems. A family‐centred approach in Prader‐Willi syndrome might benefit from considering 'family' in broad terms, such as extended family members, especially in the case of younger siblings.

### 6.1 Implications for Future Research and Practice

There is a clear need for more rigorous and detailed research into the sibling experience in the context of Prader‐Willi syndrome. Employing in‐person, child‐centred interview techniques, particularly with younger siblings, may help overcome recruitment challenges. Adopting a family systems approach, by involving multiple members of the same family, can provide deeper insight into family discourse and dynamics. A mixed‐methods design, combining in‐depth individual interviews with longitudinal approaches such as observational methods or narrative diaries, may be effective in capturing both family functioning and developmental changes over time. Recruiting a larger sample size would enhance the generalisability of findings and support the development of best practice recommendations. Encouraging parents to include siblings in discussions about family needs may positively influence both the individual with Prader‐Willi syndrome and the broader family system. Accordingly, support services should adopt a family‐centred approach to better address the holistic needs of families affected by Prader‐Willi syndrome.

## Author Contributions

**Meghana Wadnerkar Kamble:** writing (final draft, review and editing), funding acquisition, formal analysis, data curation, conceptualisation. **Jen Dawe:** writing (first draft, review and editing), formal analysis, data curation. **Karen Bunning:** writing (review and editing), formal analysis, conceptualisation.

## Conflicts of Interest

The authors declare no conflicts of interest.

## Data Availability

The data that support the findings of this study are available from the corresponding author upon reasonable request.

## Appendix A

### Semi‐Structured Interview Topic Guide for Stage 1

**Table:**

Siblings	Parents
*Objective 1. To understand their perceptions of their sibling with Prader‐Willi syndrome* Question: Tell us about your sibling with Prader‐Willi syndrome. Probe: how are they *Objective 2. Understand what their family life is with the sibling with Prader‐Willi syndrome* Question: What is it like in your house growing up? *Objective 3. To understand about their family life in relation to the challenges posed by Prader‐Willi syndrome* Question: Tell us about your experiences of living with someone with Prader‐Willi syndrome Probes: about your health needs, your behaviour adjustments, your access to food, meeting your needs, day to day family life, holidays *Objective 4. To explore what support needs they think are important*. Question: What would have helped you? Probe: what will help others in a similar situation?	*Objective 1. To understand their perceptions of their other child* Question: Tell us about your child who does not have Prader‐Willi syndrome Probe: how are they *Objective 2. Understand what their family life is with the other child* Question: What is it like in your house for the other child? *Objective 3. To understand about their family life in relation to the challenges posed by Prader‐Willi syndrome* Question: Tell us about your experiences of parenting someone with Prader‐Willi syndrome Probes: about health needs, behaviour, access to food, meeting needs of all children, day to day family life, holidays *Objective 4. To explore what support needs they think are important*. Question: What would have helped you in relation to your unaffected child. Probe: what will help others in a similar situation?
